# Gender-specific differences in mental health literacy and influencing factors among residents in Foshan City, China: a cross-sectional study

**DOI:** 10.3389/fpubh.2025.1555615

**Published:** 2025-06-09

**Authors:** Zhaosong Chu, Jianling Xie, Rui Liu, Liqin Ou, Zheng Lan, Guojun Xie

**Affiliations:** ^1^Department of Clinical Psychology, The Third People’s Hospital of Foshan, Foshan, Guangdong, China; ^2^Department of Public Health, The Third People’s Hospital of Foshan, Foshan, Guangdong, China; ^3^Xinshi Hospital of Gaoming District, Foshan, Guangdong, China

**Keywords:** mental health literacy, gender differences, influencing factors, logistic regression analysis, cross-sectional study

## Abstract

**Background:**

Mental health literacy (MHL) is essential for enhancing mental well-being and addressing mental health challenges. In China, MHL levels remain low, and studies on gender differences in MHL are limited. This study aims to explore gender-specific differences in MHL and their influencing factors among residents of Foshan City, China, to provide a theoretical basis for targeted mental health interventions.

**Methods:**

Data for this study were obtained from the 2022 Foshan City Residents’ Mental Health Survey. A comprehensive sample of 9,044 participants over 18 years old was included, collecting information on sociodemographic characteristics, lifestyle habits, health-related factors, and MHL assessments. Chi-square (*χ*^2^) tests and independent samples *t*-tests were used to analyze gender differences in MHL. Additionally, multi-factor logistic regression analysis was conducted to identify factors influencing MHL across genders.

**Results:**

The overall MHL attainment rate was 8.46%. Male residents demonstrated a significantly lower attainment rate of 6.65%, compared to 10.14% among female residents. Across specific dimensions, including mental health knowledge, attitudes, and psychological skills, males consistently exhibited lower levels of MHL than females. Multi-factor logistic regression analysis identified protective factors for male MHL, including higher educational attainment, professional technical occupations, and retirement, while depression emerged as a significant risk factor. For females, protective factors included higher education, higher monthly household income, and engaging in exercise 3 to 5 times per week. Risk factors for females included being middle-aged or older and experiencing depression.

**Conclusion:**

The MHL levels among residents of Foshan City are relatively low, with notable gender differences, particularly with males exhibiting significantly lower levels compared to females. These findings highlight the ongoing necessity to enhance the dissemination and accessibility of mental health knowledge. When developing relevant policies as well as measures, it is imperative to take into account gender differences and implement tailored programs aimed at enhancing MHL among both male and female residents.

## Introduction

According to the 2019 report published by the World Health Organization, approximately 970 million people worldwide suffer from mental disorders, accounting for 13% of the global population (1). In China, it is reported that the weighted lifetime prevalence of various mental disorders among individuals aged 18 and above stands at an alarming rate of 16.57% (2). Mental disorders consistently rank as the second leading cause of Years Lived with Disability (YLDs) globally, emphasizing the urgent need for effective intervention strategies (3).

Among the various strategies to address this challenge, enhancing public mental health literacy (MHL) is regarded as one of the key factors in promoting mental well-being and preventing mental disorders (4, 5). MHL, defined as the capacity to effectively apply knowledge, skills, and attitudes related to mental health in order to maintain psychological well-being, plays a vital role in improving mental health outcomes (6–9). It encompasses not only the recognition, management, and prevention of mental disorders, but also an understanding of how to promote and sustain positive mental health (10). Higher levels of MHL are associated with earlier detection of mental health problems, reduced stigma, and increased likelihood of seeking timely and effective support, all of which contribute to improved mental health outcomes (11, 12). However, low levels of MHL remain a global challenge (13, 14). A meta-analysis found that the recognition rates for depression (25.4%), anxiety disorders (18.2%), and schizophrenia (18.4%) among the Chinese general population are alarmingly low (15). While most respondents agreed that professional help is necessary, fewer than 40% had actually utilized mental health services, and over 60% viewed psychiatric medications as harmful (15). Similar trends have been observed in other countries, where stigma and misconceptions surrounding mental illness remain pervasive (16–18).

Gender differences in mental disorders are well-documented, with females generally exhibiting higher rates of depression, anxiety, and post-traumatic stress disorder (PTSD) (19–21), while males are more likely to suffer from substance use disorders and antisocial behaviors (22, 23). And for females, they exhibit higher prevalence rates of post-traumatic stress disorder (PTSD), depression, and anxiety disorders. A 2019 global study estimated that approximately 279.6 million people worldwide were affected by depressive disorders, including 109.2 million males and 170.4 million females (3). Anxiety disorders affected an estimated 301.4 million individuals globally, with 113.9 million males and 187.5 million females impacted (3). A recent meta-analysis also revealed that COVID-19 related fear and anxiety were more prevalent in females (24). Moreover, an epidemiological study in China found that from 1990 to 2021, the burden of depression and anxiety disorders continued to rise, with females exhibiting higher incidence rates and greater disease burden compared to males (25). These disparities are shaped by a combination of biological factors, such as hormonal differences, and sociocultural influences, including gender role expectations and exposure to violence, all of which disproportionately affect females (26).

Given the well-established association between MHL and mental health outcomes, gender differences in MHL may partly explain the observed gender disparities in mental health and mental disorders. MHL influences individuals’ ability to recognize symptoms, seek professional help, and manage psychological conditions effectively (27). These factors may contribute to the differences in how males and females experience and address mental health challenges (27).

In general, females tend to exhibit higher MHL levels than males. Prior evidence suggests that females are more likely to accurately recognize mental disorders, such as anxiety and depressive disorder (28, 29). Neuroimaging research indicates that these gender differences in recognition abilities may stem from higher neural activation in brain regions associated with emotion recognition in females (30). On the other hand, males are often less likely to seek professional help, are more prone to endorse alternative remedies, and exhibit higher levels of stigma toward mental illnesses (28, 31, 32). Cultural influences also play a significant moderating role in MHL. For instance, a study has shown that Arab males demonstrate superior knowledge, more positive attitudes, and a greater willingness to seek help for mental disorders compared to females, suggesting that gender differences in MHL are not universally consistent but rather shaped by cultural contexts (33). Despite these findings, most studies on gender differences in MHL have been conducted in Western contexts, with limited localized studies in China. Therefore, gender-specific differences in MHL within the Chinese population remain unclear.

In this study, we aim to comprehensively analyze gender differences in MHL and identify the factors that influence MHL in males and females, using data sourced from the 2022 Foshan City Residents’ Mental Health Survey. By investigating the distinct characteristics of gender differences in MHL within the Chinese context, this research endeavors to establish a foundation for formulating more targeted public health policies and mental health intervention strategies (7).

## Materials and methods

### Study population

The 2022 Foshan City Residents’ Mental Health Survey was conducted from June 2022 to October 2022. The survey participants were permanent residents of Foshan. The inclusion criteria for the survey encompassed individuals aged above 18 years, residing in Foshan for more than 6 months, and capable of understanding the questionnaire content and cooperating with the survey. Exclusion criteria included those unable to communicate effectively or comprehend the questionnaire due to cognitive impairments, mental disorders, or severe physical illnesses, as well as individuals who could not be located after at least three follow-up visits conducted at different times.

### Sample size and sampling method

The sample size was calculated using the formula: $N=\frac{\mu_{a}^{2}\times p(1-p)}{\delta^{2}}\times de\mathrm{ff}$, where the significance level of *α* = 0.05, *μ_α_* = 1.96, *p* = 2.1% [the baseline prevalence of major depressive disorder outlined in the “Healthy China Action (2019–2030)”], *δ* = 0.024 (allowable error, calculated as 0.2*p*), and *deff* = 2 (design effect). The calculated sample size resulted in an approximate value of *N* = 8,954.

The survey employed a multi-stage stratified random sampling method. In the first stage, the five districts of Foshan City were stratified based on their financial revenue and expenditure status. Within each stratum, a specific number of villages or residents’ committees were chosen, taking into account their population size, leading to a total of 89 villages or residents’ committees being selected as the primary sampling units for the survey. In the second stage, within the selected villages or residents’ committees, households that were empty, commercial establishments, or had invalid addresses were excluded. The remaining households were subsequently numbered based on their door numbers, and a systematic sampling approach was conducted to determine both the starting point and the interval. This resulted in the selection of 105 households from each village or residents’ committee. In the third stage, all eligible adult residents from the sampled households were registered, and subsequently, one individual from each household was randomly selected to participate in the survey. After securing informed consent from the selected individuals, trained investigators assisted the participants in scanning a QR code on their mobile phones to access the electronic questionnaire on the Foshan City Mental Health Service Platform. For those participants who were unable to use mobile devices, paper questionnaires or face-to-face interviews were provided as alternative methods.

### Data collection

The survey consisted mainly of socio-demographic data and questionnaire data. The former includes gender, age, education level, marital status, occupation, income, exercise frequency, diet, smoking habits, drinking habits, as well as the presence of chronic diseases. The latter includes assessments of MHL, depression, anxiety, and insomnia.

Mental health literacy was evaluated using the National Mental Health Literacy Questionnaire (NMHLQ), which comprises three distinct sections. The first section contains judgment questions to assess participants’ knowledge of mental health. The second section includes self-assessment questions aimed at assessing their positive mindset, acquisition of mental health information, as well as awareness of mental health issues. The third section features case-based questions designed to measure participants’ recognition of mental disorders, their ability to overcome stigma, and their attitudes toward seeking professional help. The measurement standards for the three dimensions of MHL are as follows: (1) a total score of 80 or above on the judgment questions; (2) a total score of 24 or above on the self-assessment questions; (3) a total score of 28 or above on the case questions. MHL is considered attainment only when all three criteria are met. The detailed content and scoring criteria of the NMHLQ have been previously described in a study (34).

The Chinese version of the Patient Health Questionnaire-9 (PHQ-9) was utilized to assess depression. This questionnaire comprises 9 self-reported items, with each item scored on a scale from 0 to 3 points. The total score ranges from 0 to 27, and a score of 10 or above is considered indicative of current depression (35). The Chinese version of the Generalized Anxiety Disorder-7 (GAD-7) was employed to evaluate anxiety. This questionnaire includes 7 self-reported items, with each item scored from 0 to 4 points. The total score ranges from 0 to 21, with a score of 10 or above being indicative of current anxiety (36). The Chinese version of the Insomnia Severity Index (ISI) was used to assess insomnia. This questionnaire consists of 7 self-reported items, with each item scored on a scale from 0 to 4 points. The total score ranges from 0 to 28, and a score of 8 or higher is considered to indicate the presence of current insomnia (37).

### Statistical analysis

Statistical analysis was performed using SPSS version 25.0. Measurement data were presented as Mean ± SD, and comparisons between groups were conducted using independent samples *t*-test. Count data were expressed as frequencies and percentages, with inter-group comparisons conducted using chi-square tests (*χ*^2^). Initially, univariate analysis was conducted to screen for potential factors associated with MHL levels. Subsequently, multivariate logistic regression analysis was employed to identify the influencing factors of MHL. The statistical significance level was set at *α* = 0.05 (two-tailed).

## Results

### Comparison of socio-demographic data, lifestyle, and health-related factors between male and female residents

A total of 9,251 questionnaires were initially completed in this survey, but 207 of them were deemed invalid and were therefore excluded. Ultimately, 9,044 valid responses were obtained, resulting in an effective response rate of 97.76%. Among the respondents, 4,359 (48.20%) were male, while 4,685 (51.80%) were female. Table 1 displays the variations in socio-demographic characteristics and health-related factors between male and female participants.

**Table 1:** Gender differences in socio-demographic information, lifestyle, and health-related factors.

Variables	Category	Total (*N* = 9,044)	Male (*N* = 4,359)	Female (*N* = 4,685)	*χ* ^2^	*P*
		*n* (%)	*n* (%)	*n* (%)		
Residence	Urban	4,546 (50.27)	2092 (47.99)	2,454 (52.38)	17.386	**<0.001**
	Rural	4,498 (49.73)	2,267 (52.01)	2,231 (47.62)		
Age	18–45 years	5,567 (61.55)	2,779 (63.75)	2,788 (59.51)	20.372	**<0.001**
	45–65 years	2,816 (31.14)	1,259 (28.88)	1,557 (33.23)		
	≥65 years	661 (7.31)	321 (7.36)	340 (7.26)		
Educational years	≤6 years	788 (8.71)	277 (6.35)	511 (10.91)	77.445	**<0.001**
	6–12 years	4,114 (45.49)	2,132 (48.91)	1982 (42.31)		
	≥12 years	4,142 (45.8)	1950 (44.74)	2,192 (46.79)		
Marital status	Married	6,989 (77.28)	3,285 (75.36)	3,704 (79.06)	144.282	**<0.001**
	Unmarried	1,566 (17.32)	927 (21.27)	639 (13.64)		
	Widowed or divorced	489 (5.41)	147 (3.37)	342 (7.3)		
Monthly household income	≤3,500 ¥	3,705 (40.97)	1,498 (34.37)	2,207 (47.11)	157.230	**<0.001**
	3,500–9,000 ¥	4,728 (52.28)	2,506 (57.49)	2,222 (47.43)		
	≥9,000 ¥	611 (6.76)	355 (8.14)	256 (5.46)		
Occupation	Worker/Farmer	2088 (23.09)	1,130 (25.92)	958 (20.45)	183.381	**<0.001**
	Public Officer/Technician/soldier	1774 (19.62)	909 (20.85)	865 (18.46)		
	Business/Service/Logistics Support	960 (10.61)	480 (11.01)	480 (10.25)		
	Retired personnel	1,126 (12.45)	340 (7.8)	786 (16.78)		
	Others	3,096 (34.23)	1,500 (34.41)	1,596 (34.07)		
Exercise frequency	Hardly	4,364 (48.25)	2,120 (48.64)	2,244 (47.9)	7.601	0.055
	1–2 times/week	1739 (19.23)	873 (20.03)	866 (18.48)		
	3–5 times/week	904 (10)	432 (9.91)	472 (10.07)		
	Almost daily	2037 (22.52)	934 (21.43)	1,103 (23.54)		
Diet regularly	No	286 (3.16)	179 (4.11)	107 (2.28)	24.493	**<0.001**
	Yes	8,758 (96.84)	4,180 (95.89)	4,578 (97.72)		
Smoking status	No	7,160 (79.17)	2,507 (57.51)	4,653 (99.32)	2393.245	**<0.001**
	Used to smoke, now quit	309 (3.42)	298 (6.84)	11 (0.23)		
	Current regular smoker	1,575 (17.41)	1,554 (35.65)	21 (0.45)		
Alcohol consumption	No	7,960 (88.01)	3,341 (76.65)	4,619 (98.59)	1031.157	**<0.001**
	Used to drink, now quit	310 (3.43)	287 (6.58)	23 (0.49)		
	Current regular drinker	774 (8.56)	731 (16.77)	43 (0.92)		
Chronic diseases	No	6,410 (70.88)	2,948 (67.63)	3,462 (73.9)	42.939	**<0.001**
	Yes	2,634 (29.12)	1,411 (32.37)	1,223 (26.1)		
Depression	No	8,582 (94.89)	4,133 (94.82)	4,449 (94.96)	0.101	0.751
	Yes	462 (5.11)	226 (5.18)	236 (5.04)		
Anxiety	No	8,787 (97.16)	4,238 (97.22)	4,549 (97.1)	0.132	0.716
	Yes	257 (2.84)	121 (2.78)	136 (2.9)		
Insomnia	No	7,383 (81.63)	3,611 (82.84)	3,772 (80.51)	8.161	**0.004**
	Yes	1,661 (18.37)	748 (17.16)	913 (19.49)		

Significant results (*p* < 0.05) are highlighted in bold type.

No statistically significant differences were observed between males and females in terms of exercise frequency, anxiety levels, or depression. However, compared to their male counterparts, females were found to have a higher proportion of urban residents, individuals in the middle-age bracket, and retirees. Conversely, males were found to have a higher proportion of individuals with a moderate level of education, an unmarried status, and a higher average monthly household income. Furthermore, gender differences in lifestyle were noted, with females exhibiting healthier habits. Specifically, a higher proportion of females had regular dietary patterns and lower rates of smoking and drinking compared to males. Conversely, males were more likely to report chronic diseases, while females reported a higher incidence of insomnia (Table 1).

### Comparison of mental health knowledge between male and female residents

Out of the 20 judgment questions assessing mental health knowledge, 11 items demonstrated a correct response rate exceeding 50%, while nine items displayed a correct response rate below 50%. For items 4, 7, 13, 16, and 19, no statistically significant differences were found in the correct response rates between male and female respondents. Conversely, for the remaining 15 items, female respondents demonstrated a higher correct response rate compared to their male counterparts. Overall, these findings indicate that female participants exhibited a higher level of awareness and understanding regarding mental health knowledge compared to males (Table 2).

**Table 2:** Gender differences in mental health knowledge.

Judgment question items (correct answer)	Total (*N* = 9,044)	Male (*N* = 4,359)	Female (*N* = 4,685)	*χ* ^2^	*P*
	*n* (%)	*n* (%)	*n* (%)		
Causes and prevention of mental illness					
1. Proper exercise can reduce anxiety, depression and other mental psychological problems. √	8,598 (95.07)	4,113 (94.36)	4,485 (95.73)	9.100	**0.003**
2. The main cause of most psychosomatic abnormal problems is genetic. ×	4,551 (50.32)	2,104 (48.27)	2,447 (52.23)	14.183	**<0.001**
5. Increased social activity in older adults can help slow the decline of brain function. √	8,656 (95.71)	4,144 (95.07)	4,512 (96.31)	8.451	**0.004**
10. Watching photos or videos of car accidents and disaster scenes may cause psychological trauma. √	6,796 (75.14)	3,146 (72.17)	3,650 (77.91)	39.772	**<0.001**
11. Sustained stress has little impact on psychosocial well-being than a sudden traumatic blow. ×	3,953 (43.71)	1832 (42.03)	2,121 (45.27)	9.659	**0.002**
Symptoms and recognition of mental illness					
14. Having a cleanliness problem is obsessive compulsive disorder.×	3,316 (36.67)	1,512 (34.69)	1804 (38.51)	14.182	**<0.001**
17. Using the online psychological questionnaire, you can diagnose whether you have a mental illness. ×	3,093 (34.2)	1,361 (31.22)	1732 (36.97)	33.133	**<0.001**
18. Whether a person has mental illness or not is easy to see. ×	3,816 (42.19)	1749 (40.12)	2067 (44.12)	14.780	**<0.001**
19. If the medical examination is normal, but you always suspect that you have a disease, it may be a mental illness. √	7,305 (80.77)	3,495 (80.18)	3,810 (81.32)	1.904	0.168
Treatment of mental illness					
7. Proactively confronting things or situations that trigger anxiety can help gradually reduce anxiety problems. √	7,779 (86.01)	3,725 (85.46)	4,054 (86.53)	2.173	0.140
8. The sooner psychiatric disorders are treated, the better. √	8,767 (96.94)	4,186 (96.03)	4,581 (97.78)	23.264	**<0.001**
9. Psychosomatic disorders can be alleviated and even recovered when treated effectively. √	8,449 (93.42)	4,026 (92.36)	4,423 (94.41)	15.395	**<0.001**
20. When a psychiatric disorder gets better with medication, you can reduce the amount of medication while observing it yourself. ×	3,021 (33.4)	1,202 (27.58)	1819 (38.83)	128.490	**<0.001**
Mind–body health associations					
15. Bad emotions may trigger physical diseases. √	8,346 (92.28)	3,960 (90.85)	4,386 (93.62)	24.351	**<0.001**
16. Hypertension, coronary heart disease, and stomach ulcers are all psychosomatic diseases. √	5,003 (55.32)	2,413 (55.36)	2,590 (55.28)	0.005	0.944
Child protection and education					
3. Excessive stress and lack of exercise in children are detrimental to brain development. √	7,789 (86.12)	3,688 (84.61)	4,101 (87.53)	16.200	**<0.001**
4. To develop your child’s self-confidence, you should always praise your child for being smart. ×	677 (7.49)	331 (7.59)	346 (7.39)	0.141	0.707
Other basic knowledge and principles					
6. Emotions such as anxiety and restlessness are harmful. ×	1,125 (12.44)	509 (11.68)	616 (13.15)	4.488	**0.034**
12. People who suffer from insomnia at night should catch up on their sleep during the day. ×	3,796 (41.97)	1731 (39.71)	2065 (44.08)	17.672	**<0.001**
13. Drinking a small amount of alcohol before bedtime can help improve the quality of sleep. ×	2,962 (32.75)	1,440 (33.04)	1,522 (32.49)	0.308	0.579

Significant results (*p* < 0.05) are highlighted in bold type.

### Comparison of MHL between male and female residents

The overall attainment rate of MHL among residents in the study was a mere 8.46%. Specifically, male residents had a significantly lower rate of 6.65% compared to the 10.14% observed among female residents. Furthermore, females had significantly higher attainment rates than males in several domains, including mental health knowledge, mental health attitude, mental health skills, as well as correct recognition rates for depression and social anxiety (all *p* < 0.05) (Table 3).

**Table 3:** Gender differences in the attainment rate of MHL and subscale scores for each dimension.

Attainment rate	Total (*N* = 9,044)	Male (*N* = 4,359)	Female (*N* = 4,685)	Statistics	
	*n* (%)	*n* (%)	*n* (%)	*χ* ^2^	*P*
Mental health literacy attainment rate	765 (8.46)	290 (6.65)	475 (10.14)	35.435	**<0.001**
Mental health knowledge attainment rate	1,020 (11.28)	413 (9.47)	607 (12.96)	27.354	**<0.001**
Mental health attitude attainment rate	8,492 (93.9)	4,064 (93.23)	4,428 (94.51)	6.476	**0.011**
Mental health skills attainment rate	4,977 (55.03)	2,197 (50.4)	2,780 (59.34)	72.876	**<0.001**
Correct recognition rate of depressive disorders	2,511 (27.76)	1,165 (26.73)	1,346 (28.73)	4.52	**0.033**
Correct recognition rate of social anxiety disorder	5,911 (65.36)	2,686 (61.62)	3,225 (68.84)	51.947	**<0.001**

Scores	(Mean ± SD)	(Mean ± SD)	(Mean ± SD)	*t*	*P*
Mental health literacy	116.69 ± 19.31	114.54 ± 19.79	118.69 ± 18.63	−10.251	**<0.001**
Knowledge of mental health	59.60 ± 15.01	58.12 ± 15.27	60.97 ± 14.64	−9.075	**<0.001**
Positive mental attitude	10.30 ± 1.96	10.39 ± 1.95	10.22 ± 1.97	4.193	**<0.001**
Access to mental health information	6.95 ± 1.09	6.98 ± 1.11	6.93 ± 1.07	2.556	**0.011**
Mental health consciousness	11.45 ± 1.07	11.39 ± 1.14	11.51 ± 0.98	−5.28	**<0.001**
Mental illness recognition	9.63 ± 4.19	9.52 ± 4.23	9.73 ± 4.16	−2.346	**0.019**
Psychological professional help seeking attitudes	6.79 ± 1.74	6.75 ± 1.78	6.83 ± 1.69	−2.273	**0.023**
Overcoming self-stigma of illness	2.43 ± 1.49	2.25 ± 1.51	2.60 ± 1.45	−11.21	**<0.001**
Overcoming the stigma of illness	9.53 ± 2.84	9.13 ± 3.00	9.89 ± 2.65	−12.756	**<0.001**

MHL, mental health literacy; SD, standard deviation.

Significant results (*p* < 0.05) are highlighted in bold type.

Comparative analyses of scores on various subscales revealed that female residents performed significantly better than males in total MHL, mental health knowledge, mental health awareness, recognition of mental disorders, attitudes toward seeking professional psychological assistance, overcoming self-stigma, as well as general stigma (all *p* < 0.05). In contrast, male residents scored significantly higher in areas including maintaining a positive mental attitude and accessing mental health information (all *p* < 0.05) (Table 3).

### Regression analysis of influencing factors on MHL levels among male and female residents

Univariate analysis revealed statistically significant differences in MHL levels among residents based on factors such as household registration types, age, marital status, occupation, exercise frequency, smoking status, drinking status, presence of chronic diseases, and depression (as displayed in Supplementary Table S1). These statistically significant factors were subsequently included as independent variables, with the attainment of MHL serving as the dependent variable (0 = non-attainment, 1 = attainment). Further analysis was conducted using multivariate logistic regression.

For male residents, the results of the logistic regression analysis demonstrated that higher education (≥12 years of education, OR = 2.217), engaging in professional technical work (OR = 1.767), and being retired (OR = 3.18) were protective factors for the attainment of MHL. In contrast, depression emerged as a risk factor (OR = 0.298) for the attainment of MHL (Table 4).

**Table 4:** Multivariate logistic regression analysis of factors influencing MHL levels among male residents.

Variables	Category	*B*	SE	Wald	*P*	OR (95%CI)
Age	18–45 years					1
	45–65 years	−0.013	0.184	0.005	0.943	0.987 (0.688–1.416)
	≥65 years	−0.407	0.424	0.925	0.336	0.665 (0.29–1.526)
Educational years	≤6 years					1
	6–12 years	0.048	0.353	0.019	0.891	1.05 (0.525–2.098)
	≥12 years	0.796	0.373	4.563	**0.033**	2.217 (1.068–4.602)
Marital status	Married					1
	Unmarried	0.154	0.154	1.001	0.317	1.166 (0.863–1.576)
	Widowed or divorced	−0.994	0.591	2.829	0.093	0.37 (0.116–1.179)
Monthly household income	≤3,500 ¥					1
	3,500–9,000 ¥	−0.039	0.146	0.072	0.789	0.962 (0.722–1.281)
	≥9,000 ¥	−0.176	0.246	0.513	0.474	0.838 (0.518–1.358)
Occupation	Worker/Farmer					1
	Public Officer/Technician/Soldier	0.569	0.201	8.013	**0.005**	1.767 (1.191–2.62)
	Business/Service/Logistics Support	0.373	0.242	2.37	0.124	1.452 (0.903–2.333)
	Retired personnel	1.157	0.342	11.42	**0.001**	3.18 (1.626–6.221)
	Others	0.22	0.19	1.336	0.248	1.246 (0.858–1.808)
Exercise frequency	Hardly					1
	1–2 times/week	−0.127	0.163	0.604	0.437	0.881 (0.64–1.212)
	3–5 times/week	−0.012	0.208	0.003	0.955	0.988 (0.658–1.485)
	Almost daily	−0.059	0.179	0.108	0.743	0.943 (0.664–1.338)
Smoking status	No					1
	Used to smoke, now quit	−0.309	0.299	1.068	0.301	0.734 (0.408–1.32)
	Current regular smoker	−0.235	0.151	2.432	0.119	0.79 (0.588–1.062)
Alcohol consumption	No					1
	Used to drink, now quit	−0.069	0.307	0.05	0.823	0.934 (0.512–1.704)
	Current regular drinker	0.089	0.185	0.233	0.629	1.093 (0.761–1.569)
Chronic diseases	No					1
	Yes	−0.252	0.151	2.776	0.096	0.777 (0.578–1.045)
Depression	No					1
	Yes	−1.211	0.461	6.904	**0.009**	0.298 (0.121–0.735)

MHL, mental health literacy; CI, confidence interval; OR, odds ratio; SE, standard error.

Significant results (*p* < 0.05) are highlighted in bold type.

For female residents, the logistic regression analysis results indicated that higher education (≥12 years of education, OR = 1.780), being unmarried (OR = 1.383), having a higher monthly household income (3500–9,000 ¥, OR = 1.299; ≥9,000 ¥, OR = 1.953), and engaging in exercise frequency of 3–5 times per week (OR = 1.568) were protective factors for the attainment of MHL. Conversely, the middle-aged and older age groups (45–64 years, OR = 0.488; ≥65 years, OR = 0.020) and the presence of depression (OR = 0.557) emerged as risk factors for the attainment of MHL among female residents (Table 5).

**Table 5:** Multivariate logistic regression analysis of factors influencing MHL levels among female residents.

Variables	Category	*B*	SE	Wald	*P*	OR (95%CI)
Age	18–45 years					1
	45–65 years	−0.718	0.185	14.972	**<0.001**	0.488 (0.339–0.702)
	≥65 years	−0.905	0.388	5.432	**0.020**	0.404 (0.189–0.866)
Educational years	≤6 years					1
	6–12 years	−0.292	0.270	1.163	0.281	0.747 (0.44–1.269)
	≥12 years	0.577	0.287	4.041	**0.044**	1.78 (1.015–3.122)
Marital status	Married					1
	Unmarried	0.324	0.126	6.576	**0.010**	1.383 (1.079–1.772)
	Widowed or divorced	0.083	0.236	0.123	0.726	1.086 (0.684–1.725)
Monthly household income	≤3,500 ¥					1
	3,500–9,000 ¥	0.262	0.119	4.851	**0.028**	1.299 (1.029–1.641)
	≥9,000 ¥	0.669	0.199	11.306	**0.001**	1.953 (1.322–2.886)
Occupation	Worker/Farmer					1
	Public Officer/Technician/Soldier	0.006	0.174	0.001	0.974	1.006 (0.715–1.414)
	Business/Service/Logistics Support	−0.146	0.210	0.486	0.486	0.864 (0.573–1.303)
	Retired personnel	0.475	0.245	3.751	0.053	1.608 (0.994–2.599)
	Others	0.074	0.159	0.215	0.643	1.076 (0.789–1.468)
Exercise frequency	Hardly					1
	1–2 times/week	−0.003	0.131	0.001	0.981	0.997 (0.771–1.29)
	3–5 times/week	0.450	0.155	8.391	**0.004**	1.568 (1.157–2.126)
	Almost daily	−0.137	0.165	0.690	0.406	0.872 (0.631–1.205)
Smoking status	No					1
	Used to smoke, now quit	−18.837	11493.859	0	0.999	0
	Current regular smoker	0.725	0.689	1.105	0.293	2.064 (0.535–7.967)
Alcohol consumption	No					1
	Used to drink, now quit	−0.93	1.062	0.767	0.381	0.395 (0.049–3.163)
	Current regular drinker	−0.367	0.654	0.314	0.575	0.693 (0.192–2.499)
Chronic diseases	No					1
	Yes	−0.17	0.14	1.469	0.225	0.844 (0.641–1.111)
Depression	No					1
	Yes	−0.584	0.27	4.676	**0.031**	0.557 (0.328–0.947)

MHL, mental health literacy; CI, confidence interval; OR, odds ratio; SE, standard error.

Significant results (*p* < 0.05) are highlighted in bold type.

## Discussion

This study investigates gender differences in MHL using survey data collected from residents of Foshan City, China, in 2022. The results indicate that the overall attainment rate of MHL among Foshan City residents is relatively low, at only 8.46%. Specifically, the attainment rate among males is 6.65%, while it is slightly higher among females, at 10.14%. These values are far below the 20% target set by the Healthy China Initiative (2019–2030) for 2022, underscoring the urgent need for population-wide interventions to improve MHL across all groups. While females demonstrated relatively higher MHL levels than males, both groups remain well below the national benchmark, highlighting that low MHL is a widespread issue rather than one confined to a particular gender. Therefore, policymakers should adopt a “universal coverage with targeted reinforcement” approach – implementing broad-based measures, such as media campaigns and curriculum reforms, to increase MHL levels among the general population, while also designing targeted interventions to support gender-specific groups.

Regarding the three dimensions of MHL, the highest attainment rate is observed in mental health attitudes (assessed through self-assessment questions), with both male and female residents achieving over 90% in this area. The attainment rate for mental skills (case questions) is the second highest, with both genders achieving over 50%. However, the attainment rate for mental health knowledge (judgment questions) is the lowest, with male residents scoring 9.47% and female residents achieving 12.96%. These patterns align with previous research findings (34), indicating that although the public demonstrates a high level of awareness and a positive attitude toward mental health, a deficiency in comprehensive mental health knowledge hinders the translation of this awareness into practical skills and behavior. The lack of knowledge regarding mental health appears to be a crucial obstacle hindering the overall improvement of MHL.

An examination of the 20 judgment questions related to mental health knowledge (Table 2) indicates that both male and female respondents achieved a correct response rate below 50% for nine of the items. This implies that a significant number of residents possess misunderstandings regarding certain aspects of mental health knowledge. The statement “To develop your child’s self-confidence, you should always praise your child for being smart” exhibited the highest incidence of incorrect responses. This indicates a deficiency in understanding the suitable methods for fostering the psychological development of children and adolescents. Overpraising a child’s intelligence can cultivate a dependency on external affirmation, thereby undermining the cultivation of a growth mindset by diminishing the importance of personal endeavor. To address the issue, it is imperative to bolster the dissemination of mental health knowledge for children and adolescents at the societal, educational, and family levels. Additionally, the eight other items with substantial error rates were related to mental disorders. These included statements such as “Emotions like anxiety and restlessness are harmful” and “Drinking a small amount of alcohol before bedtime can help improve the quality of sleep.” These discoveries underscore that residents persist in holding misconceptions about common mental health conditions, their diagnosis, and their treatment. In light of these findings, it is essential to intensify public education efforts and disseminate precise mental health knowledge through diverse platforms, such as school syllabi, community engagements, and media outreach programs (11, 38). Additionally, there is an urgent requirement to enhance the education and training of mental health competencies to comprehensively raise the general standard of MHL (39).

In alignment with previous studies conducted on Western demographics (40–42), the results of this study reveal that female residents in Foshan, China, have higher levels of MHL than males. Specifically, female residents demonstrate superior knowledge and awareness of mental health compared to males. Additionally, females demonstrate more favorable outcomes in recognizing mental disorders, attitudes toward seeking professional psychological assistance, and overcoming stigma, including self-stigma. Conversely, male residents score higher in maintaining a positive mindset and in accessing mental health information compared to females. This finding partially aligns with previous research while demonstrating some heterogeneity (14, 28, 33, 43). The discrepancies observed in these findings may be attributed to variations in the assessment tools employed and the cultural backgrounds of the participants across various studies. Several factors can provide explanations for the observed gender differences. Firstly, females may inherently possess a heightened level of psychological awareness, introspection, and emotional sensitivity, which enhances their capacity to seek psychological assistance when confronted with mental health challenges (44). In contrast, males typically exhibit lesser capabilities in recognizing and expressing emotions, posing challenges in acknowledging and articulating their mental health concerns (45, 46). Moreover, traditional societal norms in China impose heightened expectations on males to embody “strength” and discourage the display of vulnerability, leading to an increased tendency to suppress emotions and a reluctance to seek assistance for mental health concerns. This aligns with males’ reduced ability to overcome both societal stigma and self-stigma. Lastly, males generally exhibit superior stress management skills, enabling them to maintain a more positive outlook when confronted with adverse events (47). Collectively, these results indicate that female residents possess a higher level of MHL than males, highlighting the pressing necessity for targeted mental health education initiatives aimed specifically at male residents to improve their MHL.

The results of the logistic regression analysis unveil distinct protective and risk factors that influence the MHL of male and female residents. For male residents, the protective factors include possessing a higher level of education, being employed in dedicated employment, and being retired, while depression stands out as the sole risk factor. For female residents, the protective factors encompass having a higher level of education, being unmarried, possessing a higher monthly family income, and engaging in moderate exercise frequency (3–5 times per week). In contrast, the risk factors for females include being within the middle-aged to older adult age range and experiencing depression. These findings are in alignment with previous studies which have demonstrated that individuals with higher education levels generally possess greater MHL (48, 49). This correlation is likely attributable to their exposure to more comprehensive education, which fosters an enhanced understanding of the significance of mental health, empowering them to actively acquire relevant knowledge and seek professional assistance. Furthermore, male residents who are engaged in professional technical occupations (such as public officer, technician, or soldier) and those who are retired demonstrate higher levels of MHL. This is likely due to their higher educational attainment, income levels, access to structured mental health education, psychological resilience training, as well as a greater availability of mental health resources. This finding underscores that employment significantly promotes psychological health, particularly for males (50, 51). For females, the status of being unmarried emerged as a protective factor for MHL, which aligns with the findings of previous research endeavors (52). In traditional Chinese culture, females are frequently expected to fulfill caregiving roles, encompassing responsibilities such as nurturing children, caring for older adult family members, and managing household duties. These societal expectations can impose substantial mental health burdens on married females, frequently leading them to prioritize the needs of their family over their own mental health. Consequently, married females may overlook self-care, resulting in a decline in their MHL. In contrast, unmarried females frequently enjoy greater economic independence and interpersonal freedom, which may contribute to higher levels of mental health awareness and understanding. Moreover, females with higher monthly family incomes were observed to have superior levels of MHL in comparison to those with lower incomes. This reflects the importance of economic independence in fostering improved mental health and enhancing MHL among females (53, 54). Interestingly, moderate exercise (3–5 times per week) emerged as a protective factor for female residents, potentially due to females’ heightened concerns regarding body image and overall health. Previous research has indicated that both a lack of exercise and excessive exercise frequency can lead to adverse mental health outcomes, which is consistent with our findings (55, 56).

In both males and females, recent depression was found to be associated with low levels of MHL, aligning with previous research findings (57, 58). This relationship can be attributed to the adverse effects of depression on an individual’s emotional and cognitive functions, often manifesting as symptoms such as difficulty concentrating and impaired decision-making abilities (59). These impairments obstruct the capacity to acquire knowledge and skills related to mental health, ultimately leading to decreased levels of MHL. Previous studies have demonstrated that individuals exhibiting significant depressive symptoms are only half as likely to accurately recognize their own depression compared to those with mild depressive symptoms. Moreover, individuals who accurately identify their depression are five times more likely to seek professional help than those who fail to do so (60). These findings emphasize the critical importance of early identification and treatment of depression, coupled with the provision of mental health education and support services, in bolstering the MHL of individuals experiencing depression. In addition, previous research indicates that MHL is a significant predictor of an individual’s mental health status. A positive correlation exists between higher levels of MHL and improved mental health outcomes (61). Conversely, low levels of MHL are closely linked to depression (58, 60, 62), indicating that improving MHL could act as an effective early intervention strategy for addressing mental health issues. These findings highlight the intricate relationship between MHL and depression, suggesting a pressing need for further exploration in future research. Finally, being middle-aged or older adult was identified as a risk factor for female MHL, potentially due to the generally lower levels of educational attainment within this demographic. The reintroduction of the college entrance examination in China in 1977, combined with the implementation of 9 years of compulsory education in 1986, led to comparatively lower levels of education among older individuals in comparison to younger generations. Additionally, traditional gender biases favoring males have historically constrained educational prospects for females, which may provide further insight into why this phenomenon is particularly evident among females.

### Limitations

This study is also subject to certain limitations. Firstly, the cross-sectional design employed in this research fails to unveil the dynamic characteristics of MHL over time, and it does not establish causal relationships among the influencing factors. Therefore, it is imperative for future large-scale, high-quality longitudinal studies to conduct a more thorough exploration of these relationships. Secondly, despite the numerous factors that can impact MHL, this study only examined a limited subset of common factors due to constraints in the initial research design, neglecting other potential variables such as perceived stress and social support (60, 63, 64). Future research should incorporate a wider array of relevant factors to comprehensively evaluate their influence on residents’ MHL. Thirdly, significant differences were observed between male and female participants in terms of demographic and health-related variables, such as residence, age, marital status, and employment. This imbalance in baseline characteristics may have biased the results of gender comparisons. Future studies should consider employing gender-stratified sampling or propensity score matching to improve comparability between groups. Finally, the participants in the study were all from Foshan City, Guangdong Province, China, making this study less nationally representative.

## Conclusion

In summary, this study reveals critically low levels of MHL among Foshan residents (8.46% overall attainment), with particularly severe deficits in mental health knowledge being the primary barrier to improving MHL. The observed gender differences (male: 6.65% vs. female: 10.14%), although statistically significant, should be interpreted in the broader context of universally inadequate MHL levels across all demographics. Intervention strategies must have a dual focus: population-wide interventions to address systemic knowledge gaps, such as mental health education and popular science initiatives, and targeted approaches for high-risk subgroups. For males, priority populations include those with limited education, unemployment status, or depressive symptoms. For females, at-risk groups include the less educated, married women, low-income earners, physically inactive, middle-aged to older adult and those experiencing depression. The development of targeted, population-specific interventions, coupled with the strengthening of educational initiatives aimed at improving mental health knowledge and skills, is essential to promote a comprehensive improvement in MHL.

## Data Availability

The original contributions presented in the study are included in the article/Supplementary material, further inquiries can be directed to the corresponding author.

## References

[1] WHO. (2022). World mental health report: transforming mental health for all. Available online at: https://www.who.int/publications/i/item/9789240049338 (accessed January 4, 2025)

[2] Huang Y Wang Y Wang H Liu Z Yu X Yan J. Prevalence of mental disorders in China: a cross-sectional epidemiological study. Lancet Psychiatry. (2019) 6:211–24. doi: 10.1016/s2215-0366(18)30511-x, PMID: 30792114

[3] Collaborators GMD. Global, regional, and national burden of 12 mental disorders in 204 countries and territories, 1990-2019: a systematic analysis for the global burden of disease study 2019. Lancet Psychiatry. (2022) 9:137–50. doi: 10.1016/s2215-0366(21)00395-3 35026139 PMC8776563

[4] Yeo G Reich SM Liaw NA Chia EYM. The effect of digital mental health literacy interventions on mental health: systematic review and Meta-analysis. J Med Internet Res. (2024) 26:e51268. doi: 10.2196/51268, PMID: 38421687 PMC10941000

[5] Haeri-Mehrizi A Mohammadi S Rafifar S Sadighi J Kermani RM Rostami R. Health literacy and mental health: a national cross-sectional inquiry. Sci Rep. (2024) 14:13639. doi: 10.1038/s41598-024-64656-7, PMID: 38871848 PMC11176292

[6] Jiang G Zhao C Wei H Yu L Li D Lin X. Mental health literacy: connotation, measurement and new framework. J Psychol Sci. (2020) 43:232–8. doi: 10.16719/j.cnki.1671-6981.20200132

[7] Kutcher S Wei Y Coniglio C. Mental health literacy: past, present, and future. Can J Psychiatr. (2016) 61:154–8. doi: 10.1177/0706743715616609, PMID: 27254090 PMC4813415

[8] Bjørnsen HN Eilertsen MEB Ringdal R Espnes GA Moksnes UK. Positive mental health literacy: development and validation of a measure among Norwegian adolescents. BMC Public Health. (2017) 17:717. doi: 10.1186/s12889-017-4733-6 28923031 PMC5604188

[9] Jorm AF. Why we need the concept of "mental health literacy". Health Commun. (2015) 30:1166–8. doi: 10.1080/10410236.2015.1037423, PMID: 26372027

[10] Soria-Martínez M Navarro-Pérez CF Pérez-Ardanaz B Martí-García C. Conceptual framework of mental health literacy: results from a scoping review and a Delphi survey. Int J Ment Health Nurs. (2024) 33:281–96. doi: 10.1111/inm.13249, PMID: 37921340

[11] Jorm AF. Mental health literacy: empowering the community to take action for better mental health. Am Psychol. (2012) 67:231–43. doi: 10.1037/a0025957, PMID: 22040221

[12] Jorm AF Barney LJ Christensen H Highet NJ Kelly CM Kitchener BA. Research on mental health literacy: what we know and what we still need to know. Aust N Z J Psychiatry. (2006) 40:3–5. doi: 10.1080/j.1440-1614.2006.01734.x, PMID: 16403031

[13] Ganasen KA Parker S Hugo CJ Stein DJ Emsley RA Seedat S. Mental health literacy: focus on developing countries. Afr J Psychiatry. (2008) 11:23–8. doi: 10.4314/ajpsy.v11i1.30251, PMID: 19582321

[14] Tay JL Tay YF Klainin-Yobas P. Mental health literacy levels. Arch Psychiatr Nurs. (2018) 32:757–63. doi: 10.1016/j.apnu.2018.04.007, PMID: 30201205

[15] Li W Reavley N. Recognition and beliefs about treatment for mental disorders in mainland China: a systematic review and meta-analysis. Soc Psychiatry Psychiatr Epidemiol. (2020) 55:129–49. doi: 10.1007/s00127-019-01799-3, PMID: 31641829

[16] Tambling RR D'Aniello C. Mental health literacy and relational health literacy among college students. J Am Coll Heal. (2025) 73:1–9. doi: 10.1080/07448481.2023.2228428 37399581

[17] Rafal G Gatto A DeBate R. Mental health literacy, stigma, and help-seeking behaviors among male college students. J Am Coll Heal. (2018) 66:284–91. doi: 10.1080/07448481.2018.1434780, PMID: 29419361

[18] Kaneko Y Motohashi Y. Male gender and low education with poor mental health literacy: a population-based study. J Epidemiol. (2007) 17:114–9. doi: 10.2188/jea.17.114, PMID: 17641446 PMC7058472

[19] Haering S Schulze L Geiling A Meyer C Klusmann H Schumacher S. Higher risk-less data: a systematic review and meta-analysis on the role of sex and gender in trauma research. J Psychopathol Clin Sci. (2024) 133:257–72. doi: 10.1037/abn0000899 38619461

[20] Salk RH Hyde JS Abramson LY. Gender differences in depression in representative national samples: Meta-analyses of diagnoses and symptoms. Psychol Bull. (2017) 143:783–822. doi: 10.1037/bul0000102, PMID: 28447828 PMC5532074

[21] Gao W Ping S Liu X. Gender differences in depression, anxiety, and stress among college students: a longitudinal study from China. J Affect Disord. (2020) 263:292–300. doi: 10.1016/j.jad.2019.11.121, PMID: 31818792

[22] McHugh RK Votaw VR Sugarman DE Greenfield SF. Sex and gender differences in substance use disorders. Clin Psychol Rev. (2018) 66:12–23. doi: 10.1016/j.cpr.2017.10.012 29174306 PMC5945349

[23] O’Dell C Charles NE Barry CT. Gender differences in links between antisocial features and forms and functions of aggression among at-risk youth. J Psychopathol Behav Assess. (2024) 46:357–72. doi: 10.1007/s10862-024-10134-3

[24] Metin A Erbiçer ES Şen S Çetinkaya A. Gender and COVID-19 related fear and anxiety: a meta-analysis. J Affect Disord. (2022) 310:384–95. doi: 10.1016/j.jad.2022.05.036, PMID: 35561885 PMC9090872

[25] Tian W Yan G Xiong S Zhang J Peng J Zhang X. Burden of depressive and anxiety disorders in China and its provinces, 1990-2021: findings from the global burden of disease study 2021. Br J Psychiatry. (2025):1–11. doi: 10.1192/bjp.2024.267, PMID: 39838265

[26] Johnson BE. Gender differences in mental health. Salem Press Encyclopedia of Health [Preprint]. (2025). Available at: http://research.ebsco.com/linkprocessor/plink?id=3515775b-f78d-372e-a529-f6de161ae4a7 (Accessed May 26, 2025).

[27] Lee HY Hwang J Ball JG Lee J Yu Y Albright DL. Mental health literacy affects mental health attitude: is there a gender difference? Am J Health Behav. (2020) 44:282–91. doi: 10.5993/ajhb.44.3.1, PMID: 32295676

[28] Cotton SM Wright A Harris MG Jorm AF McGorry PD. Influence of gender on mental health literacy in young Australians. Aust N Z J Psychiatry. (2006) 40:790–6. doi: 10.1080/j.1440-1614.2006.01885.x 16911755

[29] Haavik L Joa I Hatloy K Stain HJ Langeveld J. Help seeking for mental health problems in an adolescent population: the effect of gender. J Ment Health. (2019) 28:467–74. doi: 10.1080/09638237.2017.1340630, PMID: 28719230

[30] Derntl B Finkelmeyer A Eickhoff S Kellermann T Falkenberg DI Schneider F. Multidimensional assessment of empathic abilities: neural correlates and gender differences. Psychoneuroendocrinology. (2010) 35:67–82. doi: 10.1016/j.psyneuen.2009.10.006, PMID: 19914001

[31] Schroeder S Tan CM Urlacher B Heitkamp T. The role of rural and urban geography and gender in community stigma around mental illness. Health Educ Behav. (2021) 48:63–73. doi: 10.1177/1090198120974963, PMID: 33218261

[32] Alluhaibi BA Awadalla AW. Attitudes and stigma toward seeking psychological help among Saudi adults. BMC Psychol. (2022) 10:216. doi: 10.1186/s40359-022-00923-4, PMID: 36109773 PMC9479300

[33] Bener A Ghuloum S. Gender differences in the knowledge, attitude and practice towards mental health illness in a rapidly developing Arab society. Int J Soc Psychiatry. (2011) 57:480–6. doi: 10.1177/0020764010374415, PMID: 20591923

[34] He XY Tan WY Guo LL Ji YY Jia FJ Wang SB. Mental health literacy among urban and rural residents of Guangdong Province, China. Risk Manag Healthc Policy. (2024) 17:2305–18. doi: 10.2147/rmhp.S479868 39371938 PMC11451470

[35] Levis B Benedetti A Thombs BD. Accuracy of patient health questionnaire-9 (PHQ-9) for screening to detect major depression: individual participant data meta-analysis. BMJ. (2019) 365:1476. doi: 10.1136/bmj.l1476 PMC6454318 30967483

[36] Sapra A Bhandari P Sharma S Chanpura T Lopp L. Using generalized anxiety Disorder-2 (GAD-2) and GAD-7 in a primary care setting. Cureus. (2020) 12:e8224. doi: 10.7759/cureus.8224, PMID: 32582485 PMC7306644

[37] Wong ML Lau KNT Espie CA Luik AI Kyle SD Lau EYY. Psychometric properties of the sleep condition Indicator and insomnia severity index in the evaluation of insomnia disorder. Sleep Med. (2017) 33:76–81. doi: 10.1016/j.sleep.2016.05.019, PMID: 28449911

[38] Lee JE Goh ML Yeo SF. Mental health awareness of secondary schools students: mediating roles of knowledge on mental health, knowledge on professional help, and attitude towards mental health. Heliyon. (2023) 9:e14512. doi: 10.1016/j.heliyon.2023.e14512, PMID: 36950622 PMC10025912

[39] Murthy MKS Kapanee ARM Desai G Chaturvedi SK. Exploring the knowledge and attitude of public about mental health problems: a pilot intervention for effective mental health promotion. J Educ Health Promot. (2019) 8:177. doi: 10.4103/jehp.jehp_137_19, PMID: 31867362 PMC6796309

[40] Gorczynski P Sims-Schouten W. Evaluating mental health literacy amongst US college students: a cross sectional study. J Am Coll Heal. (2024) 72:676–9. doi: 10.1080/07448481.2022.2063690, PMID: 35471990

[41] Chakraverty D Baumeister A Aldin A Seven ÜS Monsef I Skoetz N. Gender differences of health literacy in persons with a migration background: a systematic review and meta-analysis. BMJ Open. (2022) 12:e056090. doi: 10.1136/bmjopen-2021-056090, PMID: 37667874 PMC9301804

[42] Gibbons RJ Thorsteinsson EB Loi NM. Beliefs and attitudes towards mental illness: an examination of the sex differences in mental health literacy in a community sample. Peer J. (2015) 3:e1004. doi: 10.7717/peerj.1004, PMID: 26413429 PMC4581769

[43] Andrews KL Jamshidi L Shields RE Teckchandani TA Afifi TO Fletcher AJ. Examining mental health knowledge, stigma, and service use intentions among Royal Canadian Mounted Police cadets. Front Psychol. (2023) 14:1123361. doi: 10.3389/fpsyg.2023.1123361 37205089 PMC10187145

[44] Petrides KV Furnham A Martin GN. Estimates of emotional and psychometric intelligence: evidence for gender-based stereotypes. J Soc Psychol. (2004) 144:149–62. doi: 10.3200/socp.144.2.149-162, PMID: 15074503

[45] Wingenbach TSH Ashwin C Brosnan M. Sex differences in facial emotion recognition across varying expression intensity levels from videos. PLoS One. (2018) 13:e0190634. doi: 10.1371/journal.pone.0190634, PMID: 29293674 PMC5749848

[46] Donges US Kersting A Suslow T. Women's greater ability to perceive happy facial emotion automatically: gender differences in affective priming. PLoS One. (2012) 7:e41745. doi: 10.1371/journal.pone.0041745, PMID: 22844519 PMC3402412

[47] Tommasi M Sergi MR Picconi L Saggino A. The location of emotional intelligence measured by EQ-i in the personality and cognitive space: are there gender differences? Front Psychol. (2022) 13:985847. doi: 10.3389/fpsyg.2022.985847 36687855 PMC9846219

[48] Al-Qerem W Jarab A Khdour M Eberhardt J Alasmari F Hammad A. Assessing mental health literacy in Jordan: a factor analysis and Rasch analysis study. Front Public Health. (2024) 12:1396255. doi: 10.3389/fpubh.2024.1396255, PMID: 39011325 PMC11248750

[49] Snow KSR Merrill K Macintosh J Thomas M Miles L. Mental health literacy in Polynesian native Hawaiian and other Pacific islanders. Int J Ment Health Nurs. (2024) 33:683–92. doi: 10.1111/inm.13275, PMID: 38071505

[50] Drake RE Wallach MA. Employment is a critical mental health intervention. Epidemiol Psychiatr Sci. (2020) 29:e178. doi: 10.1017/s2045796020000906, PMID: 33148366 PMC7681163

[51] Padrosa E Vanroelen C Muntaner C Benach J Julià M. Precarious employment and mental health across European welfare states: a gender perspective. Int Arch Occup Environ Health. (2022) 95:1463–80. doi: 10.1007/s00420-022-01839-7, PMID: 35142869

[52] Xiuzhen S Jie L Yi X Jingmin Z Linghui Z. Mental health literacy among residents in Jiaxing City. J Prev Med. (2023) 35:911–5. doi: 10.19485/j.cnki.issn2096-5087.2023.10.018

[53] Shekhawat K Sharma P Menaria P. Financial independence and maternal mental health-a right balance. Southeast Asian J Health Prof. (2022) 5:4–7. doi: 10.18231/j.sajhp.2022.002

[54] Mellar BM Fanslow JL Gulliver PJ McIntosh TKD. Economic abuse by an intimate partner and its associations with women's socioeconomic status and mental health. J Interpers Violence. (2024) 39:4415–37. doi: 10.1177/08862605241235140 39380255 PMC11465629

[55] Golshani S Najafpour A Hashemian SS Goudarzi N Shahmari F Golshani S. When much is too much-compared to light exercisers, heavy exercisers report more mental health issues and stress, but less sleep complaints. Healthcare. (2021) 9:1289. doi: 10.3390/healthcare9101289 34682969 PMC8535876

[56] Senaratne N Stubbs B Werneck AO Stamatakis E Hamer M. Device-measured physical activity and sedentary behaviour in relation to mental wellbeing: an analysis of the 1970 British cohort study. Prev Med. (2021) 145:106434. doi: 10.1016/j.ypmed.2021.106434, PMID: 33485998

[57] Zhong SL Wang SB Ding KR Tan WY Zhou L. Low mental health literacy is associated with depression and anxiety among adults: a population-based survey of 16, 715 adults in China. BMC Public Health. (2024) 24:2721. doi: 10.1186/s12889-024-20020-y, PMID: 39370527 PMC11456243

[58] Lam LT. Mental health literacy and mental health status in adolescents: a population-based survey. Child Adolesc Psychiatry Ment Health. (2014) 8:26. doi: 10.1186/1753-2000-8-26

[59] Bredemeier K Berenbaum H Brockmole JR Boot WR Simons DJ Most SB. A load on my mind: evidence that anhedonic depression is like multi-tasking. Acta Psychol. (2012) 139:137–45. doi: 10.1016/j.actpsy.2011.11.007, PMID: 22154348 PMC3249471

[60] Kim JE Saw A Zane N. The influence of psychological symptoms on mental health literacy of college students. Am J Orthopsychiatry. (2015) 85:620–30. doi: 10.1037/ort0000074, PMID: 26052815 PMC4658251

[61] Song J Feng K Zhang D Wang S Wang W Li Y. The relationship between mental health literacy, overall adaptation and mental health of university freshers. Psychol Res Behav Manag. (2023) 16:4935–47. doi: 10.2147/prbm.S437718, PMID: 38089531 PMC10711296

[62] Huang X Wang X Hu J Xue Y Wei Y Wan Y. Inadequate mental health literacy and insufficient physical activity potentially increase the risks of anxiety and depressive symptoms in Chinese college students. Front Psych. (2021) 12:753695. doi: 10.3389/fpsyt.2021.753695, PMID: 34867541 PMC8637166

[63] Li S Sheng Y Jing Y. How social support impact teachers' mental health literacy: a chain mediation model. Front Psychol. (2022) 13:851332. doi: 10.3389/fpsyg.2022.851332, PMID: 35369149 PMC8968130

[64] Qian G Wu Y Wang W Lei R Zhang W Jiang S. Perceived stress and mental health literacy among Chinese preschool teachers: a moderated mediation model of anxiety and career resilience. Psychol Res Behav Manag. (2023) 16:3777–85. doi: 10.2147/prbm.S422311, PMID: 37720171 PMC10503560

