# Supplementary material for: Gender-specific differences in mental health literacy and influencing factors among residents in Foshan City, China: a cross-sectional study
Source: Front Public Health. 2025 Jun 9;13:1555615. doi: 10.3389/fpubh.2025.1555615 (PMC12183028; doi:10.3389/fpubh.2025.1555615)
Supplement: Supplementary file 1 [file Table_1.DOCX]

**Table S1**

Univariate Analysis of Factors Influencing MHL Among Residents of Foshan City

| Variables | Category | Component ration | Attainment rate | χ^2^ | *P* |
| --- | --- | --- | --- | --- | --- |
|  |  | n (%) | n (%) |  |  |
| Residence | Urban | 4546 (50.27) | 406 (8.93) | 2.633 | 0.105 |
|  | Rural | 4498 (49.73) | 359 (7.98) |  |  |
| Age | 18-45 years | 5567(61.55) | 598(10.74) | 97.563 | **<0.001** |
|  | 45-65 years | 2816(31.14) | 137(4.87) |  |  |
|  | ≥65 years | 661(7.31) | 30(4.54) |  |  |
| Educational years | ≤6 years | 788(8.71) | 35(4.44) | 213.493 | **<0.001** |
|  | 6-12 years | 4114(45.49) | 187(4.55) |  |  |
|  | ≥12 years | 4142(45.8) | 543(13.11) |  |  |
| Marital status | Married | 6989 (77.28) | 542 (7.76) | 45.419 | **<0.001** |
|  | Unmarried | 1566 (17.32) | 197 (12.58) |  |  |
|  | Widowed or divorced | 489(5.41) | 26(5.32) |  |  |
| Monthly household income | ≤3500 ¥ | 3705(40.97) | 236(6.37) | 38.502 | **<0.001** |
|  | 3500-9000 ¥ | 4728(52.28) | 457(9.67) |  |  |
|  | ≥9000 ¥ | 611(6.76) | 72(11.78) |  |  |
| Occupation | Worker/Farmer | 2088 (23.09) | 113 (5.41) | 75.944 | **<0.001** |
|  | Public Officer/Technician/soldier | 1774 (19.62) | 223 (12.57) |  |  |
|  | Business/Service/Logistics Support | 960 (10.61) | 78 (8.13) |  |  |
|  | Retired personnel | 1126 (12.45) | 66 (5.86) |  |  |
|  | Others | 3096 (34.23) | 285 (9.21) |  |  |
| Exercise frequency | Hardly | 4364(48.25) | 398(9.12) | 29.447 | **<0.001** |
|  | 1-2 times/week | 1739(19.23) | 153(8.8) |  |  |
|  | 3-5 times/week | 904(10) | 98(10.84) |  |  |
|  | almost daily | 2037(22.52) | 116(5.69) |  |  |
| Diet regularly | No | 286(3.16) | 24(8.39) | 0.002 | 0.967 |
|  | Yes | 8758(96.84) | 741(8.46) |  |  |
| Smoking status | No | 7160 (79.17) | 669 (9.34) | 34.913 | **<0.001** |
|  | Used to smoke, now quit | 309 (3.42) | 14 (4.53) |  |  |
|  | Current regular smoker | 1575 (17.41) | 82 (5.21) |  |  |
| Alcohol consumption | No | 7960 (88.01) | 705 (8.86) | 14.178 | **0.001** |
|  | Used to drink, now quit | 310 (3.43) | 14 (4.52) |  |  |
|  | Current regular drinker | 774 (8.56) | 46 (5.94) |  |  |
| Chronic diseases | No | 6410 (70.88) | 613 (9.56) | 34.677 | **<0.001** |
|  | Yes | 2634 (29.12) | 152 (5.77) |  |  |
| Depression | No | 8582 (94.89) | 744 (8.67) | 9.628 | **0.002** |
|  | Yes | 462 (5.11) | 21 (4.55) |  |  |
| Anxiety | No | 8787 (97.16) | 748 (8.51) | 1.161 | 0.281 |
|  | Yes | 257 (2.84) | 17 (6.61) |  |  |
| Insomnia | No | 7383 (81.63) | 628 (8.51) | 0.117 | 0.733 |
|  | Yes | 1661 (18.37) | 137 (8.25) |  |  |

Abbreviations: MHL mental health literacy.

Notes: Significant results (p < 0.05) are highlighted in bold type.
